# Detection and Genome Sequencing of SARS-CoV-2 in a Domestic Cat with Respiratory Signs in Switzerland

**DOI:** 10.3390/v13030496

**Published:** 2021-03-17

**Authors:** Julia Klaus, Marina L. Meli, Barbara Willi, Sarah Nadeau, Christian Beisel, Tanja Stadler, ETH SARS-CoV-2 Sequencing Team, Herman Egberink, Shan Zhao, Hans Lutz, Barbara Riond, Nina Rösinger, Hanspeter Stalder, Sandra Renzullo, Regina Hofmann-Lehmann

**Affiliations:** 1 Clinical Laboratory, Vetsuisse Faculty, Department of Clinical Diagnostics and Services, and Center for Clinical Studies, University of Zurich, Winterthurerstrasse 260, 8057 Zurich, Switzerland; mmeli@vetclinics.uzh.ch (M.L.M.); hlutz@vetclinics.uzh.ch (H.L.); briond@vetclinics.uzh.ch (B.R.); rhofmann@vetclinics.uzh.ch (R.H.-L.); 2 Clinic for Small Animal Internal Medicine, Vetsuisse Faculty, University of Zurich, Winterthurerstrasse 260, 8057 Zurich, Switzerland; bwilli@vetclinics.uzh.ch (B.W.); nina.roesinger@uzh.ch (N.R.); 3 Department of Biosystems Science and Engineering, ETH Zurich, 4058 Basel, Switzerland; sarah.nadeau@bsse.ethz.ch (S.N.); christian.beisel@bsse.ethz.ch (C.B.); tanja.stadler@bsse.ethz.ch (T.S.); 4 SIB Swiss Institute of Bioinformatics, 4058 Basel, Switzerland; 5 Department of Biomolecular Health Sciences, Faculty of Veterinary Medicine, University of Utrecht, 3584 CL Utrecht, The Netherlands; H.F.Egberink@uu.nl (H.E.); s.zhao@uu.nl (S.Z.); 6 Institute for Virology and Immunology IVI, Sensemattstrasse 293, 3147 Mittelhäusern, Switzerland; hanspeter.stalder@vetsuisse.unibe.ch (H.S.); sandra.renzullo@ivi.admin.ch (S.R.); 7 Department of Infectious Diseases and Pathobiology, Vetsuisse Faculty, University of Bern, 3001 Bern, Switzerland

**Keywords:** SARS-CoV-2, COVID-19, domestic cat, companion animals, next generation sequencing, serology, neutralizing activity, human-to-feline transmission, contamination, One-Health

## Abstract

Since the emergence of coronavirus disease (COVID-19) in late 2019, domestic cats have been demonstrated to be susceptible to severe acute respiratory syndrome coronavirus-2 (SARS-CoV-2) under natural and experimental conditions. As pet cats often live in very close contact with their owners, it is essential to investigate SARS-CoV-2 infections in cats in a One-Health context. This study reports the first SARS-CoV-2 infection in a cat in a COVID-19-affected household in Switzerland. The cat (Cat 1) demonstrated signs of an upper respiratory tract infection, including sneezing, inappetence, and apathy, while the cohabiting cat (Cat 2) remained asymptomatic. Nasal, oral, fecal, fur, and environmental swab samples were collected twice from both cats and analyzed by RT-qPCR for the presence of SARS-CoV-2 viral RNA. Both nasal swabs from Cat 1 tested positive. In addition, the first oral swab from Cat 2 and fur and bedding swabs from both cats were RT-qPCR positive. The fecal swabs tested negative. The infection of Cat 1 was confirmed by positive SARS-CoV-2 S1 receptor binding domain (RBD) antibody testing and neutralizing activity in a surrogate assay. The viral genome sequence from Cat 1, obtained by next generation sequencing, showed the closest relation to a human sequence from the B.1.1.39 lineage, with one single nucleotide polymorphism (SNP) difference. This study demonstrates not only SARS-CoV-2 infection of a cat from a COVID-19-affected household but also contamination of the cats’ fur and bed with viral RNA. Our results are important to create awareness that SARS-CoV-2 infected people should observe hygienic measures to avoid infection and contamination of animal cohabitants.

## 1. Introduction

In late December 2019, a human respiratory disease of unknown origin emerged in the province of Hubei, China. The causative agent, a novel coronavirus (CoV), named severe acute respiratory syndrome coronavirus-2 (SARS-CoV-2) was identified shortly after [1,2,3,4]. The disease, which is now referred to as coronavirus disease 2019 (COVID-19), spread rapidly among humans and was declared as a pandemic by the World Health Organization (WHO) in March 2020 [5]. This pandemic led to 75 million registered infections and 1.66 million deaths worldwide within the first year and is still ongoing [6,7]. In Switzerland, the SARS-CoV-2 infection rates primarily spiked in March 2020, followed by a second and larger wave in October and November 2020, with a total of 320,000 infections confirmed by the end of November 2020 [8]. However, in Switzerland, human-to-animal transmission of SARS-CoV-2 had not been described until now.

SARS-CoV-2 is taxonomically classified among the genus of Betacoronaviruses, in the family of Coronaviridae [2]. CoV are large, single-stranded, positive-sense RNA viruses with a genome size up to 33 kb, and they show high mutation rates upon replication [9]. Due to the low fidelity of the viral RNA polymerase and the occurrence of natural homologous recombination, CoV are versatile and highly variable viruses.

An example of viral recombination can be found in feline coronaviruses (FCoV), where the FCoV serotype II originated from a recombination event of FCoV serotype I and a canine coronavirus, which are both Alphacoronaviruses [10]. FCoV infection occurs in cats worldwide with a prevalence of up to 80% in serological analyses. A small portion of FCoV-infected cats go on to develop feline infectious peritonitis (FIP). Viral genomic mutations and the host individual inflammatory response are assumed to support the development of this systemic fatal disease [11,12]. In human CoV, zoonotic spillovers have been demonstrated previously, especially in the recent past in the newly emerged Betacoronaviruses SARS-CoV and Middle East respiratory syndrome coronavirus (MERS-CoV) [9,13,14,15]. For the moment, the origin of SARS-CoV-2 is unclear, although sequence comparisons of the viral genome to other CoV known in bats and pangolins have shown close relations [3,16,17].

For cell entry, SARS-CoV-2 spike proteins bind to the host angiotensin converting enzyme-2 (ACE2) receptor, which is a common cell membrane protein in many mammalian species [18,19]. The feline and human ACE2 were found to have 85.2% identity, which is the highest identity of an ACE in companion animals in relation to the human ACE protein [20]. In addition to cats, other animal species were also found to be susceptible to SARS-CoV-2. Under natural conditions, domestic cats, large wild felids, dogs, mink, and ferrets have reportedly been infected presumably through close contact with SARS-CoV-2 positive humans [21,22,23,24,25,26].

In experimental settings, viral replication has been detected in rhesus and cynomolgus macaques, domestic cats, ferrets, and golden hamsters; moreover, transmission of the virus from infected to cohoused animals was demonstrated [27,28,29,30,31,32,33,34,35]. Reports have also documented respiratory or gastrointestinal signs in cats after natural SARS-CoV-2 infection [21,23,36,37,38] and pathological changes in the respiratory tract in experimental studies [28]. The severity of disease in naturally SARS-CoV-2 infected symptomatic cats ranged from mild to severe in respiratory signs, including, e.g., dyspnea, sneezing, coughing, and ocular discharge, and mild to moderate in gastrointestinal signs, like diarrhea and vomitus [23,36,37,38]. Necropsy in one cat, although SARS-CoV-2 infected, showed that the death was caused by an underlying hypertrophic cardiomyopathy, which resulted in a pulmonary edema and thrombosis [39]. Another cat, which was euthanized due to the severity of dyspnea, was found in necropsy to have suffered from viral pneumonia, with detectable SARS-CoV-2 antigen by immunofluorescent staining of lung tissue [36].

In serological screenings, the prevalence of anti-SARS-CoV-2 antibodies in cats from Germany, Italy, Croatia, France, and China ranged from 0.69% to 23.5% [40,41,42,43,44,45]. A study from China could not detect SARS-CoV-2 specific antibodies in any of the 423 tested cat sera collected after November 2019 during the early stages of the COVID-19 pandemic [42], and a serological investigation in nine cats living with COVID-19 positive students at a veterinary campus in France could not prove SARS-CoV-2 infection in the tested cats. However, a significantly higher seroprevalence in animals that lived in COVID-19-affected households compared to the animals with unknown contact status was shown previously in a study from France [44]. Moreover, in a recent study from Texas, neutralizing antibodies were found in 41.2% of 17 cats from COVID-19-affected households [46].

Domestic pet cats often live in close contact with their owners. In Switzerland, about 30% of all households accommodate at least one cat. In 2020, the Swiss cat population counted over 1.7 million individuals [47]. Therefore, the surveillance of intra- and interspecies transmission of this novel CoV is of great importance in a One-Health perspective. This is especially true for SARS-CoV-2, which has already effectively crossed interspecies barriers.

The Clinical Laboratory at the Vetsuisse Faculty in Zurich, Switzerland, has been investigating the prevalence of SARS-CoV-2 in companion animals since March 2020, with a current focus on COVID-19-affected households during the second wave in Switzerland. Here, we report the first confirmed case of SARS-CoV-2 infection in a cat from a COVID-19-affected household in Switzerland. The positive RT-qPCR and sequencing results in a second cohoused cat could not be confirmed using serology because no blood sample was available. Our findings reinforce the reports on cats being susceptible to SARS-CoV-2 infection under natural conditions and support the assumption that SARS-CoV-2 associated disease in cats may occur.

## 2. Materials and Methods

### 2.1. Animals

**Figure 1:**
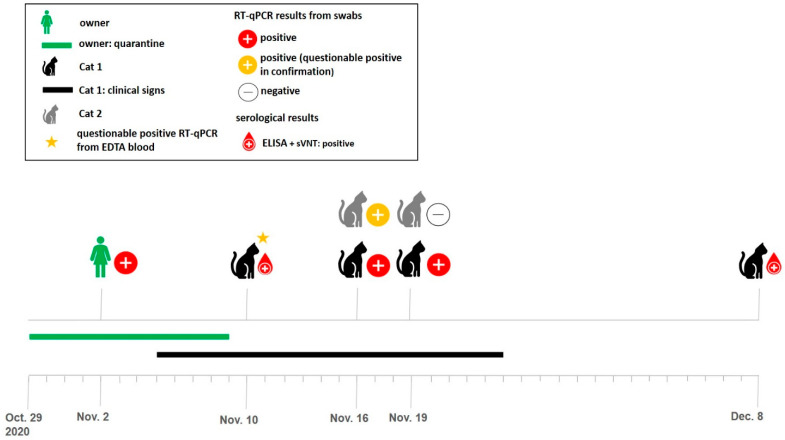
Timeline of the sample collections and overview of test results in the two cats (Cat 1 and Cat 2) after one of the two cat owners had tested RT-qPCR positive for severe acute respiratory syndrome coronavirus-2 (SARS-CoV-2). Cat 1 (indicated with the black cat-pictogram) became symptomatic on 5 November 2020 and was presented to a private practitioner on 10 November 2020, where a blood sample was collected. Swab samples from both cats were collected on 16 and 19 November 2020. Serological testing (ELISA and sVNT) was performed on serum from Cat 1 collected on 10 November and 8 December 2020. Cat 2 is indicated gray. Results of RT-qPCR and serology are indicated by colors as shown in the legend. (EDTA: ethylenediamine tetra-acetic acid, ELISA: enzyme-linked immunosorbent assay, sVNT: surrogate virus neutralization test, and RT-qPCR: real-time quantitative reverse transcriptase-polymerase chain reaction, CT: cycle threshold).

The two cats reported in this study lived in a COVID-19-affected household in the northern part of the canton of Zurich, Switzerland. The household consisted of two people living with their two cats. One owner had tested positive for SARS-CoV-2 RT-PCR on 2 November 2020 (Viollier AG, Allschwil, Switzerland; Figure 1), after both owners had developed respiratory symptoms in the week prior. The positive tested owner reported muscle pain, anosmia, cough, sore throat, and nasal discharge. The other person experienced only mild symptoms. They underwent quarantine until 9 November 2020.

The two cats in this household were four years old, male neutered, Maine Coon mixed breeds with outdoor access. One of the cats (Cat 1) developed clinical signs on 5 November 2020, during the human quarantine and was presented to a local veterinary practitioner on 10 November 2020, where a blood sample was collected.

Subsequently, the cat owners contacted the Clinical Laboratory at the Vetsuisse Faculty in Zurich, Switzerland, and the cats were enrolled into the SARS-CoV-2 surveillance study in companion animals, which is currently ongoing. For this study, mucosal, fecal, fur, and environmental swab samples were collected from both cats (Cats 1 and 2). As environmental samples, the cats’ favorite sleeping spots (fabric surfaces, each cat had one designated spot) were sampled. Further blood samples were collected from Cat 1 (Figure 1). The owner did not allow for more swab samplings.

The owners were informed about the study design and gave written consent beforehand. The sample collection was officially approved by the ethics committee of the canton of Zurich (BASEC number 2020-00979) and by the veterinary office of the canton of Zurich (ZH062/20). At the time of the cat sampling, the owners were postsymptomatic and had finished their quarantine. A veterinarian of our group, provided with personal protective equipment, sampled the cats at their home according to a sample collection protocol that was developed for the surveillance study. To prevent contamination between the two cats, gloves were changed before proceeding with the second cat.

### 2.2. Blood Sample Collection

The first blood collection from Cat 1 was performed by the local veterinarian on 10 November 2020 (Figure 1). He collected an ethylenediamine tetra-acetic acid (EDTA) and a lithium–heparin anticoagulated blood sample as well as a native blood sample for the gain of serum. Blood and serum samples were stored at 4 °C for seven days before they were made available for the current study. Subsequently, for the present study, additional EDTA and serum samples from Cat 1 were drawn on 8 December 2020 (Figure 1). The owner did not allow for any blood collection from Cat 2.

### 2.3. Swab Sample Collection

Swab sampling of both cats, Cat 1 and Cat 2, and their environment was carried out on 16 and 19 November 2020. Cotton swabs with plastic shafts (Lidl, Weinfelden, Switzerland and Heinz Herenz, Hamburg, Germany) that were separately sealed in reclosable plastic bags (Minigrip^®^ Red line, Alpharetta, GA, USA) were used for sampling. Strict caution was taken to prevent contamination of the swab sampling area during collection.

From each cat, oropharyngeal, nasal, and rectal swabs were collected. Additionally, swabs from the fur and the surface of the cats preferred sleeping spot were taken to look for environmental and surface contamination. After sampling, the swab tip was stored in a previously labeled 1.5 mL screw cap tube (Sarstedt AG and Co. KG, Nümbrecht, Germany) prefilled with 300 µL of DNA/RNA shield solution (Zymo Research Europe GmbH, Freiburg, Germany), which also ensures nucleic acid stability during sample storage/transport at ambient temperatures and inactivates nucleases and infectious agents. The overlaying part of the shaft was cut with clean scissors and the tube was closed. The specimens were shipped to the Clinical Laboratory at ambient temperature, stored at 4 °C, and further analyzed within 48 h.

### 2.4. Nucleic Acid Extraction and Molecular Analysis

The swab samples were prepared prior to extraction as previously described [48,49,50]. In detail, after mixing by vortexing, the tubes were put on a shaking incubator at 42 °C for 10 min to resuspend the sample. The incubation step was then followed by centrifugation at 8000 rpm for 1 min to remove all drops from the lid. Further preparation was carried out in a laminar flow cabinet under sterile conditions. The forceps were cleaned with RNase AWAY™ (Thermo Fisher Scientific, Basel, Switzerland) and 70% ethanol between every step and sample. The swab was inverted with a pair of sterilized forceps and the tube was centrifuged (8000 rpm for 1 min) again to retain all the liquid from the tip.

Subsequently, the swab tip was removed, and the sample was further processed for nucleic acid extraction as follows. A volume of 90 µL of total nucleic acid (TNA) was extracted from 200 µL of each sample using a MagNA Pure LC 2.0 instrument (Roche Diagnostics AG, Rotkreuz, Switzerland) with the MagNA Pure LC Total Nucleic Acid High Performance Kit (Roche Diagnostics AG) according to the manufacturer’s instructions. For each batch of extraction, a negative control (phosphate-buffered saline (PBS) without Ca^2+^ and Mg^2+^, Life Technologies Ltd., Paisley, UK) was included to monitor for cross-contamination. From the EDTA anticoagulated blood samples, RNA was extracted from 200 µL with the QIAamp RNA blood mini kit (Qiagen, Hilden, Germany) following the manufacturer’s instructions.

Two SARS-CoV-2 real-time reverse transcriptase-polymerase chain reaction (RT-qPCR) assays were used to amplify a template on the envelope gene sequence (E-assay) and the RNA dependent-RNA polymerase (RdRp-assay) on the open reading frame-1ab gene (ORF1ab) as previously described [51] with the following modifications. Both assays were run on an ABI PRISM 7500 Fast Sequence Detection System (Applied Biosystems, Foster City, CA, USA) using 4 µL of TNA and a TaqMan^®^ Fast Virus 1-Step Master Mix (Applied Biosystems, Foster City, CA, USA) in a RT-qPCR protocol designed by the Swiss Federal Institute for Virology and Immunology (IVI, Mittelhäusern, Switzerland).

The 20 µL RT-qPCR reaction for the E-assay contained 5 µL 4× Master Mix, a final concentration of 200 nM of forward primer (pWhSF-E-F21; 5′-ACA GGT ACG TTA ATA GTT AAT AGC GTA CTT CT-3′; 32 nt), 200 nM of reverse primer (pWhSF-E-R22; 5′-ACA ATA TTG CAG CAG TAC GCA CA-3′, 23 nt), and 100 nM of fluorogenic probe (pWhSF-E-P23mgb; 5′-FAM-ATC CTT ACT GCG CTT CGA-MGB-3′, 18 nt). The 20 µL RT-qPCR reaction of the RdRp-assay contained 5 µL of 4× Master Mix, a final concentration of 200 nM of forward primer (pWh-RdRp-F1; 5′-AAA TGG TCA TGT GTG GCG GT-3′; 20 nt), 200 nM of reverse primer (pWh-RdRp-R2; 5′-ATT AAC ATT GGC CGT GAC AGC T-3′, 22 nt), and 100 nM of fluorogenic probe (pWh-RdRp-P3mgb; 5′-FAM-CTC ATC AGG AGA TGC C-MGB-3′, 16 nt).

The temperature profile included a step of reverse transcription for 5 min at 50 °C, followed by the polymerase activation at 95 °C for 20 s, and 45 cycles of 3 s at 95 °C, followed by 30 s at 60 °C. Negative RT-qPCR controls (RNAse-DNase-free water, AppliChem, Darmstadt, Germany), a negative extraction control (PBS) and a positive RT-qPCR control (in vitro transcribed RNA control containing three concatenated sequences of RdRp, E, and nucleocapsid (N) SARS-Cov-2 genes: RNA_Wuhan_RdRp-E-N, provided by the IVI) were added in every run [52]. The input copy number of the samples was calculated using a 10-fold serial dilution of the positive RT-qPCR control synthetic template.

All samples were run neat and diluted 1:5 in nuclease free water to detect possible RT-qPCR inhibition. The result of the two runs, neat or 1:5 diluted of each sample, which gave the lower cycle threshold (CT)-value, was used for further interpretation. The samples were judged as positive when an amplification was detected in both assays (E-Assay and RdRp-Assay) with CT-values ≤ 38. CT-values > 38 and <45 in one or both assays were stated as questionable positive. No amplification (CT-value ≥ 45) in both or one of the two assays was interpreted as negative.

### 2.5. Confirmatiory Tests for Positive RT-qPCR Results

For confirmation of the positive RT-qPCR results from the mucosal swabs collected on 16 November 2020, the extracted TNA samples were sent to the veterinary reference laboratories of Switzerland and Germany, the Swiss Federal Institute of Virology and Immunology (Switzerland), and the Friedrich–Loeffler Institute (FLI, Greifswald–Insel Riems, Germany) [51,53]. At the IVI, the reference laboratory for SARS-CoV-2 diagnostic for animals in Switzerland, the RT-qPCR results were confirmed [51]. The sample was judged positive if all assays were positive with a CT-value ≤ 38 and questionable positive, if the CT-values were >38 to 45.

### 2.6. Next Generation Sequencing

Whole genome sequencing was conducted by the Genomics Facility Basel, Switzerland. For this, the remains of the TNA extracted from the RT-qPCR positive mucosal swabs (nasal swab from Cat 1 and oral swab from Cat 2 from 16 November 2020) were transferred to the Genomics Facility. Library preparation and whole-genome sequencing was performed as previously described [54]. Briefly, the ARCTIC v3 primer scheme was used for viral genome amplification, which results in tiled amplicons of approximately 400 bp length [55]. For optimizing the results and adapting to the low viral load, two different PCR conditions (Q5 Hot Start DNA Polymerase, New England Biolabs) were used for genome amplification.

Temperature profile for PCR condition 1: 30 s 98 °C, 35× (15 s 98 °C and 300 s 63 °C), hold 4 °C; temperature profile for PCR condition 2: 30 s 98 °C, 35× (15 s 98 °C and 300 s 65 °C), and hold 4 °C. Both amplicon sets were subsequently converted to sequencing libraries. The sequencing libraries were prepared using NEBNext Ultra II DNA library prep reagents (New England Biolabs) and NEXTFLEX UDI Barcode adaptors (PerkinElmer AG, Schwerzenbach, Switzerland). For whole genome sequencing, an Illumina MiSeq system (Illumina Switzerland GmbH, Zurich, Switzerland) with a read output of 2 × 251 bases was chosen. Quality control of the raw reads was performed with V-pipe. Following quality control, the reads were mapped to the reference genome MN908947 [56].

For phylogenetic analysis, the alignment files from the two PCR conditions were combined using SAMtools_merge [57], alignment statistics were generated using BCFtools_mpileup considering maximum 10,000 reads per site and variants were called using BCFtools_call. Variants were filtered to those where the alternate allele was the majority base using BCFtools_filter [58]. Finally, VCF_consensus_builder was used to generate a consensus sequence and mask sites with <5× coverage [59]. Lineage classification was done by using the Pangolin tool [60,61]. Sequences were aligned using MAFFT and a maximum-likelihood tree was built using IQ-TREE with an HKY + F + G4 substitution model [62,63].

### 2.7. Antibody Detection by Enzyme-Linked Immunosorbent Assay (ELISA)

The sera were tested using an in-house established enzyme-linked immunosorbent assay (ELISA), for the detection of anti-SARS-CoV-2 spike glycoprotein receptor binding domain (RBD) antibodies. The protocol was mainly based on methods described earlier [64,65,66]. In detail, the antigen, a recombinant spike glycoprotein RBD SARS-Related Coronavirus 2, Wuhan-Hu-1 with C-Terminal Histidine Tag (NR-52946, BEI Resources, Manassas, VA, USA) was boiled for 3 min at 95 °C in 0.5% sodium dodecyl sulfate (SDS, Sigma-Aldrich, Buchs, Switzerland) and diluted in 0.1 M carbonate coating buffer (Na_2_CO_3_ water free, pH 9.6) (Sigma-Aldrich) to a final concentration of 2 µg/mL of antigen and 0.0025% SDS.

A 96-well MICROLON^®^, C-bottom, medium binding plate (Greiner-Bio One, St. Gallen, Switzerland) was coated with 200 ng/well of antigen by incubating for 3 h at 37 °C and overnight at 4 °C. If not used immediately, the plates were stored at −20 °C. Before use, the plates were washed three times with ELISA wash buffer (pH 7.4, 0.15 M sodium chloride, 0.2% Tween 20, Sigma-Aldrich). Whenever washing, the plates were tapped dry before proceeding. To avoid unspecific binding, blocking was performed with 100 µL/well of 2% bovine serum albumin (BSA, Sigma-Aldrich) in P3× buffer, which contains 0.15 M sodium chloride, 1 mM Na_2_-EDTA (Titriplex^®^ III, VWR, Dietikon, Switzerland), 0.05 M Tris-base [Tris(hydroxymethyl)aminomethane, Fisher Scientific, Rheinach, Switzerland) 0.1% BSA, and 0.1% Tween 20, at 37 °C for 1 h.

After three subsequent washing steps with ELISA wash buffer, 100 µL of the diluted controls and serum samples were pipetted in duplicate. The sera were previously heat inactivated at 56 °C for 1 h, and the dilution was prepared at a ratio of 1:100 with P3x buffer. Following incubation at 37 °C for 1 h and three washing steps with ELISA wash buffer, a goat anticat immunoglobulin G (IgG) horseradish peroxidase (HRP) conjugated secondary antibody (Jackson ImmunoResearch Europe, Ely, UK) was added. The conjugate dilution was prepared as 1:3000 in P3x, and 100 µL/well was used.

After one more step of incubation for 1 h at 37 °C and another three washing steps, 100 µL of a substrate solution consisting of 0.2 M citric acid pH 4.0 (Alfa Aesar, Thermo Fisher Scientific), 2% hydrogen peroxide, and 40 mM ABTS (2.2-azino-di(3-ethylbenzthiazoline-6-sulfonic acid diammonium salt)) (Sigma-Aldrich) was pipetted in each well. The optical density (OD) values were read on a spectrophotometer (SPECTRAmax PLUS 384, Bucher Biotec AG, Basel, Switzerland) at 415 nm after the substrate was incubated for 10 min at room temperature.

Positive control sera from four SARS-CoV-2 antibody-positive field cats were kindly provided by Dr. Herman Egberink and Dr. Els Broens, Faculty of Veterinary Medicine, University of Utrecht, the Netherlands. As a negative control, a serum sample from a specified pathogen free (SPF) cat collected in 2017 was used (TVB ZH095/15 [67]). Sera submitted to the diagnostic laboratory from 24 Swiss cats for routine diagnostic purposes (remaining material) between 2 October 2017 and 4 January 2020 were run as pre-COVID-19 samples. The positive OD cutoff value was calculated at six-fold standard deviations above the mean value of reactivity of all serum samples from the pre-COVID-19 cohort [68].

### 2.8. Surrogate Virus Neutralization Test (sVNT)

For the detection of neutralizing activity against SARS-CoV-2 RBD of the spike protein, the commercially available SARS-CoV-2 Surrogate Virus Neutralization Test Kit (GenScript Inc., Piscataway, NJ, USA) was used. This test detects antibodies that block the binding of SARS-CoV-2 RBD to the angiotensin converting enzyme 2 (ACE2) receptor on a cell surface in a species-independent ELISA-like setup. The test was performed according to the manufacturer’s instructions. Briefly, the samples and controls were heat inactivated at 56 °C for 1 h, if not already performed, and diluted 1:10 with sample dilution buffer and mixed with one volume of diluted HRP-RBD solution. This mixture was incubated at 37 °C for 30 min, before adding 100 µL to the capture plate, which was precoated with ACE2 protein.

After incubating for 15 min at 37 °C, the plate was washed with washing solution four times. Thereafter, 100 µL of 3,3′,5,5′-tetramethylbenzidine (TMB) solution was added per well. The plate was then incubated in the dark at room temperature for 15 min. For stopping the reaction, 50 µL of stop solution was added to each well. The OD values were read immediately on a spectrophotometer (SPECTRAmax PLUS 384, Bucher Biotec AG) at 450 nm. Positive and negative controls, provided by the kit, were included in duplicate in every run. The assay validity was based on the OD values for positive and negative controls falling in the recommended values. The percentage of inhibition, which is dependent on the titer of anti-SARS-CoV-2 antibodies, was then calculated with the formula (1):

Inhibition (%) = (1 − OD value of sample/OD value of negative control) × 100 (1)

According to the manufacturer, the results were interpreted as positive for SARS-CoV-2 neutralizing activity when the inhibition was calculated to be ≥20%, while <20% was regarded as a negative result [69]. Four positive control sera and the 24 feline pre-COVID-19 samples described above were analyzed using the sVNT. A positive cutoff value was calculated at six-fold standard deviations above the mean value of reactivity of all serum samples from the pre-COVID-19 cohort.

### 2.9. Confirmatory Serological Tests

The serological results of Cat 1 were confirmed by the Virology Division of the Faculty of Veterinary Medicine, University of Utrecht by a virus neutralization test with a pseudotyped SARS-CoV-2 spike protein as described previously [70,71]. Additionally, the result was confirmed by the Friedrich–Loeffler Institute (FLI, Greifswald–Insel Riems, Germany) using a RBD-ELISA, an indirect immunofluorescence assay and a surrogate virus neutralization test as described previously [45,69,72].

### 2.10. Testing for Further Viral Infections

The TNA from the oral swab from Cat 1 collected on 16 November 2020, and the oral swab from Cat 2 collected on 19 November 2020 were also tested for feline calicivirus (FCV) and feline herpes virus (FHV) using previously published methods [50,73]. The serum sample collected from Cat 1 on 8 December 2020, was tested for feline leukemia virus (FeLV) p27 antigen by sandwich ELISA and for feline immunodeficiency virus (FIV) antibodies to determine FIV infection by Western blot as described [74,75,76,77,78]. The TNA from EDTA anticoagulated blood from Cat 1 was tested for FeLV provirus by qPCR as previously described [78].

### 2.11. Submission to GenBank, ProMed, and OIE

The consensus sequence of Cat 1 was submitted to GenBank (submission ID SUB9088926, GISAID ID: EPI_ISL_1005699). The cat was officially reported to the world organization for animal health (OIE) on 3 December 2020 [79] and to ProMed [80].

### 2.12. Statistical Analysis

Statistical analyses were performed using GraphPad Prism version 8.4.0 (GraphPad Software for Windows, San Diego, CA, USA).

## 3. Results

### 3.1. History and Clinical Signs

At the end of October 2020, the owners of the two cats had developed respiratory symptoms compatible with COVID-19 and one of them tested positive for SARS-CoV-2 RNA by RT-qPCR assayed in a commercial laboratory at the beginning of November 2020. The second owner was not tested for SARS-CoV-2. The positive-tested owner with strong respiratory symptoms had very close contact to Cat 1 while in quarantine. Cat 1 began to show respiratory signs three days after the owner was diagnosed with COVID-19 and was presented at a private veterinary clinic on 10 November 2020. The owners reported that the cat had been sneezing and coughing and showed apathy and inappetence at that time.

The private practitioner, to whom the cat was presented, reported slightly enlarged mandibular lymph nodes and tenderness at pharyngeal and tracheal palpation in clinical examination. The internal body temperature was not elevated (38.5 °C). The cat was diagnosed with upper respiratory tract infection and received injections of an antibiotic (amoxicillin, Betamox LA^®^, Arovet AG, Switzerland), a nonsteroidal anti-inflammatory drug (NSAID, meloxicam, Metacam^®^, Boehringer Ingelheim GmbH, Switzerland), and a long-term steroid (methylprednisolone, Depo-Medrol^®^, Zoetis Schweiz GmbH, Switzerland).

Amoxicillin clavulanic acid (Clavaseptin^®^, Vetoquinol AG, Switzerland) and the NSAID robenacoxib (Onsior^®^, Elanco Animal Health Inc., Switzerland) were prescribed for continuing the treatment at home. The overall clinical condition of Cat 1 improved slowly; however, the sneezing persisted for another two weeks. No clinical signs were observed in Cat 2. Both cats had no underlying health condition. The owners reached out to the Clinical Laboratory due to concerns for possible SARS-CoV-2 infection in their symptomatic cat.

### 3.2. RT-qPCR

The nasal swab that was collected from Cat 1 on 16 November 2020, two weeks after the owners had tested positive, resulted in RT-qPCR positive results for both genes (E and RdRp; CT-values 32.6 and 33.2; Table 1). This result was replicated in another nasal swab collected three days later with similar CT-values (Table 1). All TNA samples were run neat and 1:5 diluted to test for potential RT-qPCR inhibition. Only one TNA sample in the diluted assay gave a lower CT-value (oral swab from Cat 2 collected on 16 November 2020). In all other samples, no evidence of RT-qPCR inhibition was found.

The RT-qPCR of the EDTA anticoagulated blood sample collected from Cat 1 on 10 November 2020 yielded CT-values of 43 (E gene) and 42 (RdRp gene) and was, therefore, interpreted as questionable positive. However, the fur swabs collected at both times and the cat bedding sample from Cat 1 also tested positive with CT-values ranging from 31.8 to 36.9 (Table 1).

In the asymptomatic cat (Cat 2) the first oral swab was positive, but the subsequent sample collected three days later was negative (Table 1). In addition, the bedding swab collected at the first timepoint and the fur sample collected three days later tested RT-qPCR positive (Table 1). The fecal swabs collected from both cats tested negative twice.

The positive RT-qPCR result in the nasal swab from Cat 1 collected on 16 November 2020 was confirmed by the federal laboratory of Switzerland (IVI) with CT-values of 35 in the RdRp and both E RT-qPCR assays (triplicates). This sample also tested positive in the federal laboratory of Germany (FLI) with CT-values of 35 in RdRp assay (duplicated) and a CT-value of 40 in the E RT-qPCR assay. The oral swab from Cat 2 collected on 16 November 2020, yielded a negative result in the IVI and a questionable positive result in the RdRp RT-qPCR assay in the FLI. No further samples were tested at the IVI or FLI.

### 3.3. Next Generation Sequencing

By next generation sequencing (NGS), the nearly complete SARS-CoV-2 viral genome from the nasal swab sample from Cat 1 collected on 16 November 2020 was successfully sequenced. The viral RNA was amplified using two different PCR conditions yielding consensus sequence coverages in the third quartile of 2858 and 4433, respectively, and completeness with respect to reference sequence Wuhan-Hu-1 (MN908947) of 93% and 97% of positions with ≥5× coverage, respectively. The same PCR conditions were applied to the oral swab sample from Cat 2. From that sample a poorer sequencing yield was obtained, with consensus sequence coverages in the third quartile of 972 and 0 and genome completion of 41% and 17%, respectively.

With respect to the reference Wuhan-Hu-1, twenty-two single nucleotide polymorphisms (SNPs) were present in at least one of the four sequences obtained from Cat 1 and Cat 2 (Table 2). SNPs that were inconsistent between the sequences from the same cat were not included. One SNP was in the untranslated region 5UTR (nt 241), 10 in the open reading frame ORF1ab region (nts 1236, 3037, 3738, 7122, 9130, 14349, 14408, 16111, 18186, and 19137), three in the spike (S) gene (nts 22801, 23403, and 23580), one in the ORF6 gene (nt 27319), six in the nucleocapsid (N) gene (nts 28487, 28868, 28881, 28882, 28883, and 29091) and one in the 3UTR (nt 29706). No SNPs were found in the ORF3a, envelope (E) gene, membrane (M) gene, ORF7a gene, and ORF8 gene. Most of these SNPs (*n* = 12) are nonsynonymous (Table 2). Nineteen of the 22 SNPs, including all of the 12 nonsynonymous SNPs were present in the consensus sequence of Cat 1.

**Figure 2:**
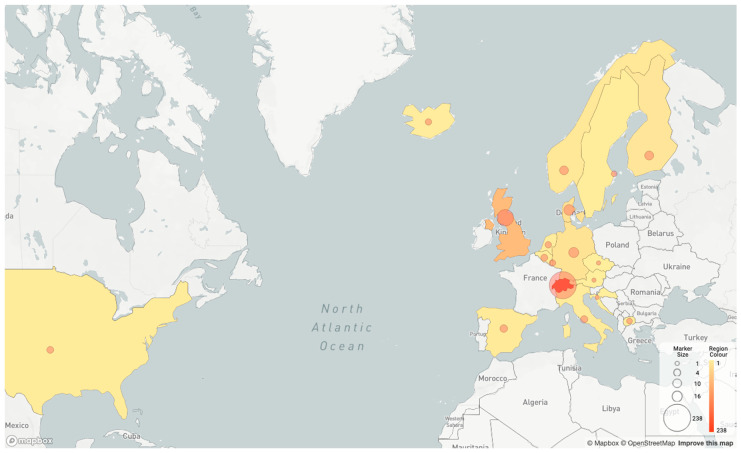
Geographic distribution of isolates from lineage B.1.1.39 according to pangolin [60]. France (2), Rwanda (1), New Zealand (1), Australia (1), Czech Republic (1), and Croatia (1) are not shown in the map generated with pangolin, but the countries reported isolates from the B.1.1.39 lineage, according to GISAID.org (isolate number in brackets).

For phylogenetic analysis, the consensus sequence of Cat 1 was considered. The specimen of the SARS-CoV-2 RT-PCR positive cat owner was no longer available in the commercial laboratory for sequence comparison. Instead, a comparison was made between the consensus sequences from Cat 1 and the sequences publicly available on the Global Initiative on Sharing All Influenza Data (GISAID) website as of 26 January 2021 [81]. The consensus sequence from Cat 1 was most similar genetically to a human-derived viral sequence (EPI_ISL_729214, Appendix A, Table A1) in the B.1.1.39 lineage. The human isolate was collected on 6 November 2020 in the same municipality as where the cat lives. With respect to the sequence of EPI_ISL_729214, the consensus sequence from Cat 1 shows one SNP (T7122C on the ORF1ab gene), while EPI_ISL_729214 shows the wildtype nucleotide “T” at this position (Table 2). The B.1.1.39 lineage was primarily found in Switzerland, where it was the second most sampled lineage in the Canton Zurich in November 2020 (geographic distribution shown in Figure 2).

Additionally, the consensus sequence of Cat 1 was compared to 18 other cat isolates available on GISAID on 26 January 2021. Four shared SNPs, with respect to the reference Wuhan-Hu-1, were found among the cat viral sequences (Table 2).

**Figure 3:**
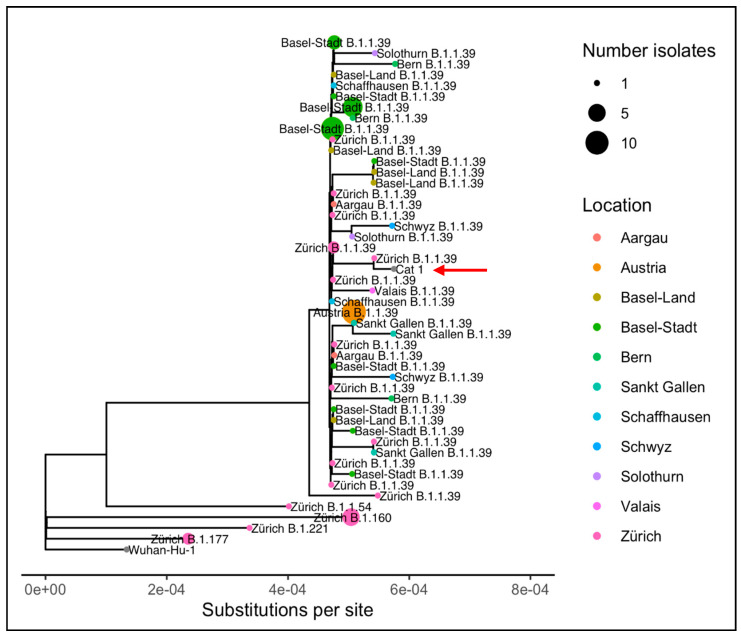
Phylogenetic tree showing the relationship between the isolate from Cat 1 and related isolates from humans (69 most genetically similar isolates from the Global Initiative on Sharing All Influenza Data (GISAID) according to nonsynonymous mutations and ten isolates representative of the predominant lineages circulating in Zurich during November 2020). Clades of isolates from the same pangolin lineage collected in the same location are collapsed [60]. The phylogenetic position of Cat 1 is indicated with the red arrow and gray dot.

A phylogenetic tree including the 69 most genetically similar isolates from GISAID according to nonsynonymous mutations and ten isolates representative of the predominant lineages circulating in Zurich in November 2020, shows that the Cat 1 consensus sequence is most closely related to the human-derived isolate from Zurich (Figure 3), although similar isolates were found across Switzerland and Austria.

### 3.4. Serological Tests

**Figure 4:**
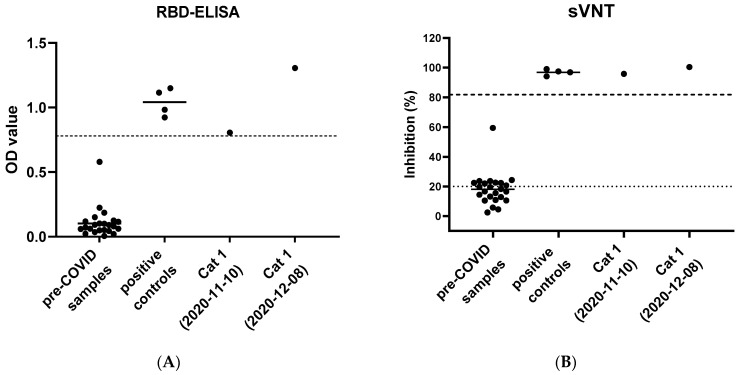
Serological results of two consecutive serum samples from Cat 1 and control samples. (**A**) receptor binding domain enzyme-linked immunosorbent assay (RBD-ELISA) results from Cat 1 compared to a precoronavirus disease 2019 (COVID-19) control group (*n* = 24) and a positive control group (*n* = 4). Serum from Cat 1 from 10 November 2020 and 8 December 2020 yielded OD values of 0.81 and 1.31, respectively. Pre-COVID-19 control group sera (collected from Swiss cats before 5 January 2020) showed a mean OD value of 0.10. Positive control group sera showed a mean OD of 1.04. The dashed line indicates six-fold standard deviations of the mean OD of the pre-COVID-19 samples. (**B**) Surrogate virus neutralization test (sVNT) results from Cat 1 compared to a pre-COVID-19 control group (*n* = 24) and a positive control group (*n* = 4). Cat 1 showed an inhibition of 95.9% and 100.4% on 10 November and 8 December 2020, respectively. The mean inhibition of pre-COVID-19 sera was calculated at 18%. Positive control sera resulted in a mean inhibition of 96.9%. The dotted line indicates the positive cutoff determined by the manufacturer at an inhibition of ≥20% The dashed line indicates six-fold standard deviations of the mean inhibition of the pre-COVID-19 samples at 81.8%.

The sera were analyzed by an in-house established ELISA for the presence of anti-SARS-CoV-2 RBD antibodies. The serum from Cat 1 collected on 10 November 2020 showed a mean OD value of 0.81 and the serum from Cat 1 from 8 December 2020, showed 1.31 as the mean OD value (Figure 4A). Pre-COVID-19 sera from 24 cats were included. Apart from one pre-COVID-19 sample with an OD of 0.580 (collected on 30 August 2019), all samples yielded low OD values (<0.225); the cutoff for positive samples was set at the six-fold standard deviation of the pre-COVID-19 samples and was an OD of 0.78 (Figure 4A). Four positive control cat serum samples resulted in a mean OD of 1.04. The sample from the negative SPF cat resulted in a mean OD of 0.05.

SARS-CoV-2 neutralizing activity was assessed using the sVNT. The first serum sample from Cat 1 (collected on 10 November 2020) showed an inhibition of 95.9% with an OD value of 0.12. The subsequent serum sample from Cat 1 (collected on 8 December 2020) showed an inhibition of 100.4% with an OD value of 0.053 (Figure 4B). Thus, inhibition in the samples from Cat 1 was clearly above the 20% inhibition judged to be positive by the manufacturer.

In addition, pre-COVID-19 sera from 24 cats were analyzed. Apart from one pre-COVID-19 sample with 59.4% inhibition (collected on 22 November 2019), all samples yielded low inhibition with a mean inhibition at 18%. If a cutoff was set at the six-fold standard deviation above the mean inhibition of the pre-COVID-19 samples (81.8% inhibition), the samples from Cat 1 were still considered positive. The four positive control samples resulted in a mean inhibition of 96.9%. No samples from Cat 2 were available for serological analyses.

Positive serological findings for the serum from Cat 1 collected on 10 November 2020 were confirmed by the Virology Division of the Faculty of Veterinary Medicine, University of Utrecht: the serum had a virus neutralization titer of 1:512; ≥16 is considered positive. The positive results of the sample were confirmed by the FLI: Cat 1 tested positive in an RBD-ELISA (OD = 0.86; positive OD ≥ 0.3), by an indirect immunofluorescence assay (titer 1:64; positive titer > 1:16) and in the surrogate virus neutralization test (88.5% inhibition; positive inhibition > 20%).

### 3.5. Exclusion of Further Common Feline Viral Infections

RT-qPCR and qPCR, respectively, from oral swabs from both cats, tested negative for FCV and FHV. Additionally, EDTA blood from Cat 1 was negative for FeLV provirus. Cat 1 also tested negative for FIV infection by FIV Western blot and for FeLV infection (antigenemia and provirus negative). Blood samples from Cat 2 were not available.

## 4. Discussion

We hereby report and describe the first case of a SARS-CoV-2 infection in a cat from a COVID-19-affected household in Switzerland. SARS-CoV-2 positive domestic cats had been reported in other European countries, i.e., Belgium [37], France [38], Spain [39,82], Germany [83], UK [36], and Italy [84], as well as in Hong Kong [25] and the US [23]. Most of these cases and SARS-CoV-2 positive cats from some additional countries appear on a list that is regularly updated by the world organization for animal health (OIE) [21]. The SARS-CoV-2 positive cats reported so far lived in COVID-19-affected households, as was the case in the current study, or lived with suspectedly SARS-CoV-2 infected humans. The authors of the current report share the opinion that the transmission of the SARS-CoV-2 infection in Cat 1 most probably occurred through close contact with infected humans. The United States Department of Agriculture had introduced a case definition for positive cases, which was implemented in the United States [85]. According to that, a confirmed positive case is defined as an animal with “SARS-CoV-2 real-time RT-PCR and sequence confirmation of virus either direct from sample or from virus isolate; or demonstration of SARS-CoV-2 neutralizing antibodies” [85]. The hereby reported case of Cat 1 meets those defined criteria.

In the present study, two four-year-old male neutered Maine Coon mixed breed cats were tested for SARS-CoV-2 infection after one of them had developed signs of respiratory disease. The presence of active SARS-CoV-2 infection could undoubtably be demonstrated in one of the cats (Cat 1) using diverse molecular and serological methods, and confirmation of our results was provided by the federal laboratory of Switzerland (IVI) and Germany (FLI), respectively, and by a research laboratory in the Netherlands (University of Utrecht). For the second cat (Cat 2), only molecular data could be provided.

In the nasal swabs from Cat 1, viral RNA was detected at two timepoints, three days apart. The first sampling was conducted eleven days after the onset of respiratory signs in this cat and two weeks after one of the owners had tested SARS-CoV-2 RT-PCR positive. Regarding the history, the timeline, and onset of clinical signs in Cat 1, human-to-cat transmission appears the most likely. As previous studies reported, under experimental conditions, positive RT-qPCR results may be expected only within a short timeframe after SARS-CoV-2 infection of cats. In one study, nasal and oral swabs were RT-qPCR positive up to day 14 after the experimental infection [33], and infectious virus could be isolated from one-day post inoculation to up to seven days after virus inoculation or contact transmission in other studies [32,35]. However, in a recent field study, viral RNA was detectable over a period of approximately 25 days in one cat [46]. Further studies will be necessary to determine the expected time range of RT-PCR positivity in pet cats under natural conditions.

With a CT-value of about 33 in the E and RdRp assay, resulting from the nasal swabs from Cat 1 at both timepoints, a viral RNA load of approximately 9000 copies per swab was calculated. In naturally infected cats, only a few reports provided details on CT-values. In Spain, two cats tested positive for SARS-CoV-2 in serological tests, while one of them was also positive in RT-qPCR from nasal swabs, with CT-values from 33 to 39 [39]. SARS-CoV-2 RNA was detected in rectal swabs collected from a cat in France that showed mild respiratory and gastrointestinal symptoms with a CT-value of 29 [38]. A cat in Italy that had shown acute signs of pneumonia, was tested positive for viral RNA with CT-values of 34 in a nasal swab [84], and a cat from the UK yielded CT-values of 34 and 33.5 in the two different genes [36]. In the case of Cat 1, it can be assumed that lower CT-values and, therefore, higher viral loads could have been found during earlier stages of the infection and the acute phase of disease. It is doubtable that, at the time of testing, Cat 1 shed infectious virus. Human studies, investigating the correlation between CT-values and virus isolation, found samples to be noninfectious starting at CT-values > 24 to ≥30 or higher [86]. In general, culturable samples had significantly lower CT-values and, therefore, higher viral loads, as summarized in a recent review that has not yet undergone peer-review [86]. Research also showed that positive viral culturing was the most likely in samples collected at early timepoints of infection [87]. Thus far, successful viral culturing was documented in a field sample from a cat in Texas and two tigers and a lion in New York. In RT-qPCR, the cats’ sample yielded CT-values ranging from 18 to 22, depending on the targeted gene [46]. The field samples from the two tigers and the lion in New York had CT-values ranging from 17.3 to 24.5 in the lowest tested targets [88]. Therefore, sample collection early in the infection, while high viral loads are expected, is crucial to gain further insight in the shedding patterns of infectious SARS-CoV-2 in animals. In addition to low viral loads, the rare reporting of viral isolation results in veterinary samples may be explained by the restriction to biosafety-level-3 (BSL-3) laboratories.

Another crucial issue in the study setup, beside the timepoint of sample collection, is the selection of the appropriate sample material for SARS-CoV-2 RNA detection in cats, as only limited information is available thus far on potential shedding routes. As reported previously, viral RNA could be retained from deep oral, nasal, and fecal swabs, as well as from vomit and ocular samples from cats [23,25,38,82]. A potential explanation for the occurrence of viral RNA in all these different materials and locations could be the expression of ACE2, the host cell receptor for SARS-CoV-2. This receptor is present in many different cell types from various organ systems in mammals [18,89]. Therefore, we suggest that sampling materials for SARS-CoV-2 RT-qPCR should be chosen broadly. In our study, oral, nasal, and fecal swabs were tested to minimize the risk of missing positive results and to gain insight in potential shedding routes of SARS-CoV-2 in cats.

Interestingly, fur samples from Cat 1 and the cats’ bedding were also RT-qPCR positive; the CT-values of these samples were similar to those of the nasal samples from Cat 1. Whether the viral RNA positive results were due to virus shedding of the cat (e.g., by sneezing) or to contamination by the cat owner cannot be determined. This was likely not due to grooming of the cat, since the oral swab from Cat 1 was RT-qPCR negative.

It is also important to note that the SARS-CoV-2 RT-qPCR protocols and the interpretation of the obtained RT-qPCR results vary between laboratories, as also shown in the present study, where the CT-values differed slightly between the three laboratories. An additional reason for slight differences in CT-values could be due to the sample shipping, although the TNA was shipped either by courier on dry ice or, for short distances, on ice packs. The interpretation of the RT-qPCR results depends, furthermore, on the question under investigation.

Caution should be used if one aims to determine whether a cat poses a potential SARS-CoV-2 infection risk; this could best be answered using virus isolation—however, due to biosafety reasons this assay is not widely available and, at least in Switzerland, not available for routine diagnostic purposes for animal samples. Besides virus isolation, alternative RT-PCR methods can also be applied to detect transcriptionally active virus by using specific primers and probes for sense and antisense RNA [90]. This alternative RT-PCR method could not be applied in the case of Cat 1 and Cat 2 due to insufficient material. More targeted and systematic research on cats in COVID-19-affected households using molecular and serological methods will be necessary to elucidate whether only low SARS-CoV-2 loads may be expected in naturally infected cats and whether questionable positive loads may, nonetheless, be associated with previous viral replication in cats. The authors of this current report share the opinion that for the definition of a SARS-CoV-2 infection serological methods, and for the determination of active infection and infectiousness ideally virus isolation is needed in addition to common RT-qPCR and sequencing results. A blood sample collected from Cat 1 during the symptomatic phase of the infection yielded only questionable positive RT-qPCR results (very high CT-values), which are consistent with the very low viral RNA loads in the peripheral blood of this cat. In a literature review on human patients, SARS-CoV-2 RNA was reported in 0–76% of the tested blood samples with a pooled estimate of 10% [91]. RNA detection in human blood samples was positive up to 20 days post symptom onset in a clinical cohort of 212 patients, and the detection of viral RNA was associated with the severity of the disease [91]. However, CT-values in that study were high (≥33.5), and viremia (the presence of replicating virus) was not confirmed in any of the positive samples [91]. In eight asymptomatic cats, none of the blood samples collected at seven timepoints within 21 days after experimental infection tested positive for viral RNA [33]. In Cat 1, the viral RNA blood loads may have been underestimated, since the initial blood sample collected by a private veterinary practitioner had been stored at 4 °C for seven days prior to analysis. Prolonged sample storage at this temperature could have led to a significant reduction and, thus, underestimation of the viral RNA blood load in Cat 1. Given the results in humans and in experimental cats so far, the presence of viremia in Cat 1 seems unlikely.

An induction of a specific antiviral immune response, with anti-SARS-CoV-2 RBD-specific antibodies and neutralizing activity was detected in sera from Cat 1. This confirms an active SARS-CoV-2 infection. The exact timepoint of the infection in Cat 1 could not be determined. However, the cat became symptomatic seven days after the owner developed respiratory symptoms and while the cat was in close contact with the owner during quarantine. Cat 1 was sleeping in the same bed with the COVID-19 diseased owner, in contrast to Cat 2, which was staying more distant. The first blood sample from Cat 1 was collected five days after the cats’ clinical signs were noticed by the owner. In an experimental study, SARS-CoV-2 infected cats had detectable neutralizing antibodies starting at seven to ten days after the virus exposure [33]. Therefore, it is reasonable to assume that Cat 1 had acquired the infection from its SARS-CoV-2 positive owner during the quarantine period. Analyzing a second blood sample collected almost one month after the initial sample, an increase in RBD-specific antibodies was observed, while the neutralizing activity rose only slightly.

These findings also support our assumption that, at the time of the initial blood collection, while the cat was symptomatic, an acute SARS-CoV-2 infection was present in Cat 1. SARS-CoV-2 as the cause of disease in Cat 1 cannot be proven undoubtedly. However, the serological findings, the absence of evidence of other viral infections (FHV, FCV, FIV, and FeLV), and the timing and the presentation of the clinical signs strengthen the assumption that the respiratory signs in Cat 1 resulted as a consequence of SARS-CoV-2 infection.

The species independent sVNT used in this study was recently evaluated for cat sera using a plaque reduction assay; a high sensitivity (98.9%) and specificity (98.8%) of the sVNT was reported for feline samples [92]. In contrast, in the present study, one sample of the feline pre-COVID cohort collected on 22 November 2019 resulted in a high inhibition of 59.4% in the sVNT (but only a mean OD value of 0.09 in the RBD-ELSA; positive cutoff at 0.78). FCoV cross-reactivity seems unlikely to be the cause of the increased inhibition in the sVNT; a low FCoV titer of 1:25 was found in this sample using an indirect immunofluorescence assay (IFA) with porcine transmissible gastroenteritis virus (TEGV) as previously described [93]. The latter is in accordance with previous results, where no cross-reactivity of the sVNT to other coronaviruses was reported [92]. The cutoff of ≥20% recommended for the sVNT by the manufacturer was, in our opinion, not optimal for feline samples. We therefore suggest setting a higher cutoff in the sVNT for cats. Using this higher cutoff, the two serum samples from Cat 1 were clearly positive in the sVNT, and this positive result was confirmed using assays for the detection of neutralizing activity in two different laboratories (FLI and University of Utrecht). Of note, one other sample from the feline pre-COVID cohort group yielded high OD values in the RBD-ELISA. This sample also did not have particularly high FCoV antibodies with a titer of 1:100 and had a clearly negative result in the sVNT with 18.3%. The presence of antibodies in cats cross-reacting with SARS-CoV-2 could also be explained by a further, yet unknown feline CoV with low virulence, which is closely related but distinct from SARS-CoV-2. Currently, the cause of the false positive signals of the two feline serum samples are under investigation. Only a limited number of studies on SARS-CoV-2 seroprevalence in cats have been performed and they have used different methods and antigens [40,41,44,45]. However, also in human cohorts, samples with presumably unspecific seroreactivity to SARS-CoV-2 antigens were reported [94]. Further serological studies using more feline samples and investigating different cat populations will be necessary. However, we recommend combining different serological approaches for the confirmation of seropositive results.

In Cat 2, the oral swab, together with the fur and environmental swab, but not the nasal and fecal swabs, were positive in RT-qPCR. Thus, in Cat 2, a possible contamination of the mouth through licking and self-grooming behavior rather than infection, at least at the tested timepoints, should be considered. In a previous study, viral RNA contamination rather than infection was suspected in two dogs that tested RT-qPCR positive in fecal or fur swabs, but did not show an antibody or neutralization response [46]. Cat 2 had less close contact to the owner, but still contact to Cat 1; thus, the RT-qPCR positivity in samples from Cat 2 may also have resulted from Cat 1. Virus transmission by direct contact and airborne transmission to bystander cats has been demonstrated under experimental conditions [28,32,33]. In testing the cats’ fur and their favorite sleeping area for viral RNA, we aimed to gain insight into potential environmental SARS-CoV-2 contamination in the cats’ surroundings and of the cats in a COVID-19-affected household. Pet cats living in COVID-19-affected households could potentially act as fomites for viral spread. Interestingly, the cats’ bedding samples gave the lowest CT-values and, thus, the highest viral RNA loads were found for the cats individually. In general, SARS-CoV-2 was proven to remain infectious on different surfaces for days; this indicates that fomite transmission could occur but is dependent on viral loads and the presence of live virus [95,96]. Thus far, virus isolation from fur samples from animals was evaluated only in one study, and no infectious virus was detected [46]. Due to biosafety issues (BSL-3 requirements), determination of the infectivity could not be attempted in the present study. The rather high viral RNA loads found in the environment of the two cats and on some of the fur samples should be considered cautiously. Strict hygienic measures while in contact with animals from COVID-19-affected households and, of course, with COVID-19 patients are essential to prevent potential transmission of the infection [24].

A whole viral genome sequence from Cat 1’s nasal swab was obtained by next generation sequencing. The sequence was most closely related to a human isolate collected in the same municipality, ten days before the sample was collected from Cat 1, which showed only one SNP difference. This human-derived isolate belongs to the lineage B.1.1.39, which was widely distributed in Switzerland at the time of sample collection in November 2020. Sequence comparison and phylogenetic analysis demonstrate the close relation and reinforce the assumption that transmissions on the human–cat interface occur and feline specific viral adaptions may not be required, as also emphasized with a sequence comparison to other feline viral sequences available on GISAID respective to the viral genome sequence of Wuhan-Hu-1. Four SNPs, shared among the cats’ isolates, were present. However, these SNPs are not unique to cats and have also been found in isolates from humans across the globe. Therefore, the case reported here and the other publicly available feline viral sequences do not give reason to assume that cat-to-cat transmissions took place under natural conditions. However, due to the unavailability of the owner’s viral sequence, the exact change of sequence at the transmission interface remains unknown. Since late 2020, a global shift to viral variants, which carry mutations which alter the fitness and infectivity of SARS-CoV-2 in humans was noticed [97]. The impact of viral variants on the susceptibility of cats and other companion animals needs to be closely monitored.

Taking account of the history, date of the onset of clinical signs, exclusion of other feline viral infections, and viral genome sequence analysis, human-to-cat transmission seems most likely in the case of Cat 1. Previous studies that reported natural SARS-CoV-2 infection in cats also suspected human-to-cat transmission and so far, no zoonotic event has been determined [21,25,36,37,39]. Until today, animal-to-human transmission of SARS-CoV-2 was only confirmed in infected mink [26,98,99,100]. The prospect of mink farms building a reservoir and spreading novel viral variants to humans did raise international concerns and led to the mandated culling of millions of minks. However, the high susceptibility of mustelids and large number of animals in dense housing conditions fueled the spread and establishment of mutations of SARS-CoV-2. By contrast, in pet cats, dense housing conditions and intense inter- or intraspecies contact outside of the household are not very common. Cat rescue and breeding facilities and cat hoarders may be exceptions to this and may, therefore, be of special interest. However, obtaining clear evidence of zoonotic transmission through pet cats will be extremely difficult, as veterinary epidemiologists have stated [101].

Apart from epidemiologic concerns including widening of the host spectrum of the virus and adding to the pool of viral replication, SARS-CoV-2 infection in cats should also be considered as an animal health issue. Therefore, COVID-19 affected patients should adhere to hygiene measures when in contact with cats as they are when in contact with uninfected persons [24,102,103]. The European Advisory Board on Cat Diseases (ABCD) recommends hygiene measures in cat-owning COVID-affected households that include “handling cats only when wearing a mask, washing hands with soap and water for at least 20 s before and after being in contact with the cats, their food, or litter box, as well as avoiding kissing pet cats or sharing food, towels, or the bed with them” [104]. This will also reduce any potential risk of cat-to-human transmission in a COVID-19-affected household. However, human-to-human transmission remains the main route of infection and the driving force in the SARS-CoV-2 pandemic. SARS-CoV-2 infected animals should never be neglected or abandoned. Further surveillance and investigation in COVID-19-affected households from a One-Health perspective will be required.

## 5. Conclusions

In conclusion, we report the first Swiss cat with SARS-CoV-2 infection, confirmed by RT-qPCR from nasal swabs targeting two genes, genome sequencing, and the detection of anti-SARS-CoV-2 RBD-antibodies and neutralizing activity. The cat was living in close contact with its COVID-19 positive owner and another cat and developed moderate respiratory signs three days after the owner tested positive with RT-qPCR. The cat had no comorbidities and the clinical signs improved within 20 days after disease onset. In the cohabiting cat, an infection could not be proven, due to the absence of blood samples for serological testing. Our findings support the testing of cats when in contact with COVID-19 positive or suspected positive humans and with pertinent clinical disease. We concluded that it is essential to investigate SARS-CoV-2 infections in pet cats, to determine potential health risks posed by the infection and to further monitor the role of cats in the pandemic in a One-Health context.

## Figures and Tables

**Table 1:** The RT-qPCR results from ethylenediamine tetra-acetic acid (EDTA) blood from Cat 1 and mucosal, fecal, fur, and environmental swabs from both cats.

Cat ID	Collection Date	Material	E-Assay (CT-Value) ^1^	RdRp-Assay (CT-Value) ^1^	Interpretation of the Results ^2^
Cat 1	10 November 2020	EDTA blood	43.2	42.2	questionable positive
	16 November 2020	oral swab	negative	36.6	negative
		**nasal swab**	**32.6**	**33.2**	**Positive**
		fecal swab	negative	44	negative
		**fur swab**	**34.4**	**34.1**	**positive**
		**bedding swab**	**31.8**	**31.7**	**positive**
	19 November 2020	oral swab	negative	negative	negative
		**nasal** **swab**	**32.5**	**32.4**	**positive**
		fecal swab	negative	negative	negative
		**fur swab**	**36.9**	**35.7**	**positive**
Cat 2	16 November 2020	**oral swab**	**37.5**	**36.2**	**positive**
		nasal swab	negative	negative	negative
		fecal swab	negative	negative	negative
		fur swab	44.9	35.8	questionable positive
		**bedding swab**	**32.9**	**33.1**	**positive**
	19 November 2020	oral swab	negative	negative	negative
		nasal swab	negative	negative	negative
		fecal swab	negative	negative	negative
		**fur swab**	**33.8**	**35.9**	**positive**

^1^ Displayed cycle threshold (CT) values indicate measurements in neat samples except for the oral swab from Cat 2 (dilution 1:5) collected on 16 November 2020. ^2^ Interpretation: positive: CT-values ≤ 38 in both assays; questionable positive: CT-values between >38 and <45 in one or both assays; and negative: CT-values ≥ 45 in one or both assays.

**Table 2:** Single nucleotide polymorphisms (SNPs) that are present in at least one of the four sequences. SNPs that were inconsistent between sequences from the same cat are not reported. Genome coordinates are with reference to sequence Wuhan-Hu-1 (MN908947). SNPs that are shared between the consensus sequence of Cat 1 and 18 other cat isolates available on GISAID on 26 January 2021 are highlighted in bold letters.

Gene	SNP (nt)	aa Change	Cat 1 PCR 1	Cat 1 PCR 2	Cat 2 PCR 1	Cat 2 PCR 2
5UTR	C241T *	noncoding	T	T	n	n
ORF1ab	A1236G *	ORF1a: D324G	G	G	n	n
ORF1ab	C3037T *	synonymous	T	T	n	n
ORF1ab	C3738T *	ORF1a: P1158L	T	T	n	n
ORF1ab	T7122C *	ORF1a: I2286T	Y (C or T)	C	n	n
ORF1ab	G9130T *	synonymous	T	T	n	n
ORF1ab	T14349C	synonymous	n	n	n	C
ORF1ab	C14408T *	ORF1b: P314L	T	T	n	T
ORF1ab	C16111T *	synonymous	T	T	Y (C or T)	n
ORF1ab	G18186T *	ORF1b: M1573I	n	T	n	n
ORF1ab	A19137G *	synonymous	G	G	n	n
S	G22801T *	synonymous	T	K (T or G)	T	n
S	A23403G *	S: D614G	n	G	G	n
S	G23580C *	S: S673T	C	C	n	n
ORF6	T27319C *	synonymous	C	n	C	n
N	G28487A *	N: V72I	A	A	A	n
N	C28868T *	N: P199S	T	T	n	n
N	G28881A *	N: R203K	A	A	n	n
N	G28882A *	N: R203K	A	A	n	n
N	G28883C *	N: G204R	C	C	n	n
N	C29091T	synonymous	n	n	T	n
3UTR	G29706T	noncoding	n	n	T	n

* SNPs that are present in the consensus sequence of Cat 1 (*n* = 19); Bold: SNPs that Cat 1 has in common with viral sequences from 18 other cats available on GISAID on 26 January 2021 (*n* = 4). Abbreviations: GISAID = Global Initiative on Sharing All Influenza Data, SNP = single nucleotide polymorphism, UTR = untranslated region, ORF = open reading frame, S = spike, N = nucleocapsid, nt = nucleotide, aa = amino acid, PCR = polymerase chain reaction, n = no SNP.

## Data Availability

All available data are presented in this manuscript.

## Appendix A

**Table A1:** Publicly available genome sequences from the Global Initiative on Sharing All Influenza Data (GISAID.org) used for bioinformatics analysis (26 January 2021).

Sequence Identifier on GISAID	Submitting Laboratory	Originating Laboratory	Analysis
EPI_ISL_536400	MRC-University of Glasgow Centre for Virus Research	Fareham Creek Veterinary Surgery	isolate from cat
EPI_ISL_487275	Department of Veterinary Pathology, University of Liege-FARAH	Department of Veterinary Pathology, University of Liege-FARAH	isolate from cat
EPI_ISL_717979	Laboratory of Biology, Department of Medicine, Democritus University of Thrace, Alexandroupolis, Greece	Laboratory of Microbiology and Infectious Diseases, Faculty of Veterinary Medicine, Aristotle University of Thessaloniki, University Campus, 541 24, Thessaloniki, Greece	isolate from cat
EPI_ISL_722300	Erasmus Medical Center	Dutch COVID-19 response team	isolate from cat
EPI_ISL_683164	Albertsen Lab, Department of Chemistry and Bioscience, Aalborg University, Denmark	Department of Virus and Microbiological Special Diagnostics, Statens Serum Institut, Copenhagen, Denmark	isolate from cat
EPI_ISL_683165	Albertsen Lab, Department of Chemistry and Bioscience, Aalborg University, Denmark	Department of Virus and Microbiological Special Diagnostics, Statens Serum Institut, Copenhagen, Denmark	isolate from cat
EPI_ISL_683166	Albertsen Lab, Department of Chemistry and Bioscience, Aalborg University, Denmark	Department of Virus and Microbiological Special Diagnostics, Statens Serum Institut, Copenhagen, Denmark	isolate from cat
EPI_ISL_759858	School of Public Health, The University of Hong Kong	School of Public Health, The University of Hong Kong	isolate from cat
EPI_ISL_873040	WHO National Influenza Centre Russian Federation	WHO National Influenza Centre Russian Federation	isolate from cat
EPI_ISL_873210	Virology, Ecole Nationale Veterinaire de Toulouse	Virology, Ecole Nationale Veterinaire de Toulouse	isolate from cat
EPI_ISL_811147	WHO National Influenza Centre Russian Federation	WHO National Influenza Centre Russian Federation	isolate from cat
EPI_ISL_437349	Institut Pasteur CIBU-ERI	Ecole nationale vétérinaire d’Alfort-laboratoire de santé animale Anses UMR 1161 de virologie ENVA-Anses-INRAE	isolate from cat
EPI_ISL_699509	Diagnostic Virology Laboratory, USDA National Veterinary Services Laboratories	Diagnostic Virology Laboratory, USDA National Veterinary Services Laboratories	isolate from cat
EPI_ISL_699507	Diagnostic Virology Laboratory, USDA National Veterinary Services Laboratories	Diagnostic Virology Laboratory, USDA National Veterinary Services Laboratories	isolate from cat
EPI_ISL_699506	Diagnostic Virology Laboratory, USDA National Veterinary Services Laboratories	Diagnostic Virology Laboratory, USDA National Veterinary Services Laboratories	isolate from cat
EPI_ISL_482820	IrsiCaixa AIDS Research Lab	Centre de Recerca en Sanitat Animal (IRTA-CReSA)	isolate from cat
EPI_ISL_483063	Virology, Ecole Nationale Veterinaire de Toulouse	unknown	isolate from cat
EPI_ISL_483064	Virology, Ecole Nationale Veterinaire de Toulouse	unknown	isolate from cat
EPI_ISL_854043	Bergthaler laboratory, CeMM Research Center for Molecular Medicine of the Austrian Academy of Sciences	Austrian Agency for Health and Food Safety (AGES)	phylogeny
EPI_ISL_854044	Bergthaler laboratory, CeMM Research Center for Molecular Medicine of the Austrian Academy of Sciences	Austrian Agency for Health and Food Safety (AGES)	phylogeny
EPI_ISL_854047	Bergthaler laboratory, CeMM Research Center for Molecular Medicine of the Austrian Academy of Sciences	Austrian Agency for Health and Food Safety (AGES)	phylogeny
EPI_ISL_854048	Bergthaler laboratory, CeMM Research Center for Molecular Medicine of the Austrian Academy of Sciences	Austrian Agency for Health and Food Safety (AGES)	phylogeny
EPI_ISL_854056	Bergthaler laboratory, CeMM Research Center for Molecular Medicine of the Austrian Academy of Sciences	Austrian Agency for Health and Food Safety (AGES)	phylogeny
EPI_ISL_854062	Bergthaler laboratory, CeMM Research Center for Molecular Medicine of the Austrian Academy of Sciences	Austrian Agency for Health and Food Safety (AGES)	phylogeny
EPI_ISL_854064	Bergthaler laboratory, CeMM Research Center for Molecular Medicine of the Austrian Academy of Sciences	Austrian Agency for Health and Food Safety (AGES)	phylogeny
EPI_ISL_854081	Bergthaler laboratory, CeMM Research Center for Molecular Medicine of the Austrian Academy of Sciences	Austrian Agency for Health and Food Safety (AGES)	phylogeny
EPI_ISL_854082	Bergthaler laboratory, CeMM Research Center for Molecular Medicine of the Austrian Academy of Sciences	Austrian Agency for Health and Food Safety (AGES)	phylogeny
EPI_ISL_854087	Bergthaler laboratory, CeMM Research Center for Molecular Medicine of the Austrian Academy of Sciences	Austrian Agency for Health and Food Safety (AGES)	phylogeny
EPI_ISL_854108	Bergthaler laboratory, CeMM Research Center for Molecular Medicine of the Austrian Academy of Sciences	Austrian Agency for Health and Food Safety (AGES)	phylogeny
EPI_ISL_603551	Department of Biosystems Science and Engineering, ETH Zurich	Viollier AG	phylogeny
EPI_ISL_603729	Department of Biosystems Science and Engineering, ETH Zurich	Viollier AG	phylogeny
EPI_ISL_603513	Department of Biosystems Science and Engineering, ETH Zurich	Viollier AG	phylogeny
EPI_ISL_737750	Department of Biosystems Science and Engineering, ETH Zurich	Viollier AG	phylogeny
EPI_ISL_857526	Swiss National Reference Centre for Influenza	Swiss National Reference Centre for Influenza	phylogeny
EPI_ISL_830757	University Hospital Basel, Clinical Bacteriology	University Hospital Basel, Clinical Virology	phylogeny
EPI_ISL_830752	University Hospital Basel, Clinical Bacteriology	University Hospital Basel, Clinical Virology	phylogeny
EPI_ISL_830762	University Hospital Basel, Clinical Bacteriology	University Hospital Basel, Clinical Virology	phylogeny
EPI_ISL_603459	Department of Biosystems Science and Engineering, ETH Zurich	Viollier AG	phylogeny
EPI_ISL_603460	Department of Biosystems Science and Engineering, ETH Zurich	Viollier AG	phylogeny
EPI_ISL_830872	University Hospital Basel, Clinical Bacteriology	University Hospital Basel, Clinical Virology	phylogeny
EPI_ISL_830879	University Hospital Basel, Clinical Bacteriology	University Hospital Basel, Clinical Virology	phylogeny
EPI_ISL_830881	University Hospital Basel, Clinical Bacteriology	University Hospital Basel, Clinical Virology	phylogeny
EPI_ISL_830882	University Hospital Basel, Clinical Bacteriology	University Hospital Basel, Clinical Virology	phylogeny
EPI_ISL_830884	University Hospital Basel, Clinical Bacteriology	University Hospital Basel, Clinical Virology	phylogeny
EPI_ISL_830888	University Hospital Basel, Clinical Bacteriology	University Hospital Basel, Clinical Virology	phylogeny
EPI_ISL_830891	University Hospital Basel, Clinical Bacteriology	University Hospital Basel, Clinical Virology	phylogeny
EPI_ISL_830901	University Hospital Basel, Clinical Bacteriology	University Hospital Basel, Clinical Virology	phylogeny
EPI_ISL_830903	University Hospital Basel, Clinical Bacteriology	University Hospital Basel, Clinical Virology	phylogeny
EPI_ISL_830906	University Hospital Basel, Clinical Bacteriology	University Hospital Basel, Clinical Virology	phylogeny
EPI_ISL_830907	University Hospital Basel, Clinical Bacteriology	University Hospital Basel, Clinical Virology	phylogeny
EPI_ISL_830914	University Hospital Basel, Clinical Bacteriology	University Hospital Basel, Clinical Virology	phylogeny
EPI_ISL_830992	University Hospital Basel, Clinical Bacteriology	University Hospital Basel, Clinical Virology	phylogeny
EPI_ISL_830993	University Hospital Basel, Clinical Bacteriology	University Hospital Basel, Clinical Virology	phylogeny
EPI_ISL_830925	University Hospital Basel, Clinical Bacteriology	University Hospital Basel, Clinical Virology	phylogeny
EPI_ISL_830928	University Hospital Basel, Clinical Bacteriology	University Hospital Basel, Clinical Virology	phylogeny
EPI_ISL_830929	University Hospital Basel, Clinical Bacteriology	University Hospital Basel, Clinical Virology	phylogeny
EPI_ISL_830998	University Hospital Basel, Clinical Bacteriology	University Hospital Basel, Clinical Virology	phylogeny
EPI_ISL_830959	University Hospital Basel, Clinical Bacteriology	University Hospital Basel, Clinical Virology	phylogeny
EPI_ISL_830965	University Hospital Basel, Clinical Bacteriology	University Hospital Basel, Clinical Virology	phylogeny
EPI_ISL_830972	University Hospital Basel, Clinical Bacteriology	University Hospital Basel, Clinical Virology	phylogeny
EPI_ISL_614899	Department of Biosystems Science and Engineering, ETH Zurich	Viollier AG	phylogeny
EPI_ISL_614901	Department of Biosystems Science and Engineering, ETH Zurich	Viollier AG	phylogeny
EPI_ISL_614902	Department of Biosystems Science and Engineering, ETH Zurich	Viollier AG	phylogeny
EPI_ISL_614904	Department of Biosystems Science and Engineering, ETH Zurich	Viollier AG	phylogeny
EPI_ISL_614906	Department of Biosystems Science and Engineering, ETH Zurich	Viollier AG	phylogeny
EPI_ISL_721846	Department of Biosystems Science and Engineering, ETH Zurich	Viollier AG	phylogeny
EPI_ISL_721853	Department of Biosystems Science and Engineering, ETH Zurich	Viollier AG	phylogeny
EPI_ISL_768144	Department of Biosystems Science and Engineering, ETH Zurich	Viollier AG	phylogeny
EPI_ISL_693893	Department of Biosystems Science and Engineering, ETH Zurich	Viollier AG	phylogeny
EPI_ISL_693894	Department of Biosystems Science and Engineering, ETH Zurich	Viollier AG	phylogeny
EPI_ISL_693936	Department of Biosystems Science and Engineering, ETH Zurich	Viollier AG	phylogeny
EPI_ISL_796492	Department of Biosystems Science and Engineering, ETH Zurich	Viollier AG	phylogeny
EPI_ISL_737782	Department of Biosystems Science and Engineering, ETH Zurich	Viollier AG	phylogeny
EPI_ISL_899802	Department of Biosystems Science and Engineering, ETH Zurich	Viollier AG	phylogeny
EPI_ISL_721921	Department of Biosystems Science and Engineering, ETH Zurich	Viollier AG	phylogeny
EPI_ISL_603423	Department of Biosystems Science and Engineering, ETH Zurich	Viollier AG	phylogeny
EPI_ISL_603490	Department of Biosystems Science and Engineering, ETH Zurich	Viollier AG	phylogeny
EPI_ISL_603499	Department of Biosystems Science and Engineering, ETH Zurich	Viollier AG	phylogeny
EPI_ISL_603509	Department of Biosystems Science and Engineering, ETH Zurich	Viollier AG	phylogeny
EPI_ISL_603514	Department of Biosystems Science and Engineering, ETH Zurich	Viollier AG	phylogeny
EPI_ISL_614967	Department of Biosystems Science and Engineering, ETH Zurich	Viollier AG	phylogeny
EPI_ISL_614971	Department of Biosystems Science and Engineering, ETH Zurich	Viollier AG	phylogeny
EPI_ISL_614974	Department of Biosystems Science and Engineering, ETH Zurich	Viollier AG	phylogeny
EPI_ISL_693792	Department of Biosystems Science and Engineering, ETH Zurich	Viollier AG	phylogeny
EPI_ISL_693838	Department of Biosystems Science and Engineering, ETH Zurich	Viollier AG	phylogeny
EPI_ISL_729237	Department of Biosystems Science and Engineering, ETH Zurich	Viollier AG	phylogeny
EPI_ISL_729214	Department of Biosystems Science and Engineering, ETH Zurich	Viollier AG	phylogeny
EPI_ISL_693948	Department of Biosystems Science and Engineering, ETH Zurich	Viollier AG	phylogeny
EPI_ISL_721950	Department of Biosystems Science and Engineering, ETH Zurich	Viollier AG	phylogeny
EPI_ISL_729029	Department of Biosystems Science and Engineering, ETH Zurich	Viollier AG	phylogeny
EPI_ISL_728979	Department of Biosystems Science and Engineering, ETH Zurich	Viollier AG	phylogeny
EPI_ISL_729071	Department of Biosystems Science and Engineering, ETH Zurich	Viollier AG	phylogeny
EPI_ISL_729074	Department of Biosystems Science and Engineering, ETH Zurich	Viollier AG	phylogeny
EPI_ISL_729078	Department of Biosystems Science and Engineering, ETH Zurich	Viollier AG	phylogeny
EPI_ISL_729084	Department of Biosystems Science and Engineering, ETH Zurich	Viollier AG	phylogeny
EPI_ISL_737401	Department of Biosystems Science and Engineering, ETH Zurich	Viollier AG	phylogeny
EPI_ISL_737919	Department of Biosystems Science and Engineering, ETH Zurich	Viollier AG	phylogeny
EPI_ISL_402125	National Institute for Communicable Disease Control and Prevention (ICDC) Chinese Center for Disease Control and Prevention (China CDC)	National Institute for Communicable Disease Control and Prevention (ICDC) Chinese Center for Disease Control and Prevention (China CDC)	phylogeny
